# Construction of a disulfidptosis-associated lncRNAs risk model to predict prognosis and immuno-infiltration analysis of lung adenocarcinoma

**DOI:** 10.12669/pjms.40.10.9025

**Published:** 2024-11

**Authors:** Jiaming Liu, Hao Nie, Wenji Du, Wei Song

**Affiliations:** 1 Jiaming Liu, Guangzhou University of Chinese Medicine, Guangzhou 510405, China; 2 Hao Nie, Guangzhou University of Chinese Medicine, Guangzhou 510405, China; 3 Wenji Du, Guangzhou University of Chinese Medicine, Guangzhou 510405, China; 4 Wei Song, Department of Endocrinology, Guangdong Provincial Hospital of Chinese Medicine, Guangzhou 510405, China

**Keywords:** Lung adenocarcinoma, Long non-coding RNAs, Disulfidptosis

## Abstract

**Objective:**

To develop a risk model based on LncRNAs associated with disulfidptosis to forecast the prognosis and assess immune infiltration of Lung adenocarcinoma (LUAD).

**Methods:**

This study employed a bioinformatics approach. The study was conducted from March 29, 2023 and concluded on July 1, 2023 at Guangzhou University of Chinese Medicine, Guangzhou, China. Transcriptomic data specific to LUAD were collected from TCGA database. Disulfidptosis-related LncRNAs were preliminarily screened using co-expression analysis, followed by screening using Lasso regression and Cox regression to identify LncRNAs. Subsequently, prognostic prediction models were constructed. To assess the model, survival analysis, subject operating characteristic curves, and calibration curves were employed. To evaluate the tumor microenvironment, the “estimate” package was used, while the “ggpubr” package was utilized to visualize the variations. Additionally, we employed CIBERSORT to examine immune cell infiltration abundance.

**Results:**

A prognostic prediction model was constructed using five LncRNAs. The high-risk group displayed a shorter overall survival and progression-free survival (P<0.05). The concordance index was calculated as 0.704 (95%

**CI:**

0.654-0.754). GSEA analysis reveals that high risk group is associated with the cell cycle pathway and steroid hormone biosynthesis pathway, while the low-risk group is associated with hematopoietic cell pathway and allograft rejection pathway. Immune cell infiltration analysis indicated associations between the prognostic model and activated T cells CD4 memory, T cells CD8, etc.

**Conclusions:**

The risk model of Disulfidptosis-related LncRNAs can predict the prognosis of LUAD and evaluate the immune infiltration, providing a new direction for the treatment of LUAD.

## INTRODUCTION

Lung cancer is a significant cause of cancer-related deaths globally and is responsible for a substantial burden on healthcare systems worldwide.^1^ Non-small-cell lung cancer (NSCLC) is the most common subtype of lung cancer, accounting for 80% of all cases. Lung adenocarcinoma (LUAD) is the predominant type of NSCLC and represents approximately half of all NSCLC diagnoses.^2^ LUAD is characterized by dense lymphocyte infiltration and is prone to metastasis at an early stage.^3^ Despite significant progress in research on molecular pathology, clinical oncology, and targeted therapy, the mortality rate remains high, with a five years survival rate of only 15% for advanced-stage patients.^4^,^5^ Therefore, the development of a more effective prognostic prediction model for LUAD is urgently needed to guide prevention of LUAD patients and improve their survival rate.

A new form of regulated cell death, known as disulfidptosis, has been discovered through recent studies.^6^ Xiaoguang Liu et al^7^ revealed that cancer cells experience limited nicotinamide adenine dinucleotide phosphate (NADPH) under conditions of glucose starvation, due to the restricted generation of NADPH from glucose through the pentose phosphate pathway (PPP). This results in the irregular accumulation of disulfides that are unable to be reduced, resulting in increase in the disulfide bond.

This triggers the contraction of actin filament (F-actin), destroying the cytoskeletal structure and eventually leading to cell death. Moreover, this particular cell death cannot be reversed while blocking other death mechanisms, which is named disulfidptosis. In addition, research has shown that disulfidptosis exists in cancer cells that express high level of SLC7A11 gene.^8^

Long non-coding RNAs (lncRNAs) are transcripts of more than 200 nucleotides in length that lack long and evolutionarily conserved open reading frames with no protein-coding capacity.^9^ In recent years, many results have been achieved in lncRNAs related to ferroptosis and cuproptosis characteristic genes. Several studies have showed that lncRNAs can assess immune cell infiltration and forecast immunotherapy efficacy in cancers such as LUAD.^10^ Tang Y et al.^11^ proved the correlation between lncRNAs related to multiple-ferroptosis characteristic genes and head and neck squamous cell carcinoma.

The above research shows that disulfidptosis-related lncRNAs may act as a promising treatment target for LUAD. In this study, our goal was to develop a LUAD prediction model of the disulfidptosis-related lncRNAs based on TCGA data and analyse utility of this model from a clinical perspective, providing new ideas and directions for the early clinical prevention of LUAD and theoretical foundation for the clinical management of LUAD.

## METHODS

This study employed a bioinformatics approach. The research design was selected based on the objectives of the study. The study was conducted over a period of three months. Data collection and data analysis commenced on March 29, 2023 and concluded on July 1, 2023. The research was carried out at Guangzhou University of Chinese Medicine, Guangzhou 510405, China.

### Ethical approval:

Not applicable.

This study aimed to collect transcriptomic data specific to LUAD and identify lncRNAs associated with disulfidptosis. Co-expression analysis, Lasso regression, and Cox regression will be used to screen and identify specific lncRNAs. Prognostic prediction models will be constructed using the identified lncRNAs, and their accuracy and reliability will be evaluated. The tumor microenvironment will be assessed using bioinformatics tools, including estimating the microenvironment and examining immune cell infiltration.

### Collection of transcriptome and clinical data of LUAD:

The Cancer Genome Atlas (TCGA) database includes multiple data of more than 30 cancers. We downloaded the transcriptome data of LUAD project from the TCGA database (TCGA-LUAD, https://portal.gdc.cancer.gov/) and utilized Perl software to organize the data. Then we extracted information of expression matrix and clinical profile by utilizing Perl software and distinguished between mRNAs and lncRNAs. The data were all downloaded on March 29, 2023. Lastly, we analyzed tumor mutational burden (TMB) and obtained tumor mutational matrix. The data we downloaded was available in an open access format and did not necessitate approval from the Medical Ethics Committee.

### Acquisition of disulfidptosis genes:

The disulfidptosis genes were derived from recent reports, including GYS1, NDUFS1, OXSM, LRPPRC, NDUFA11, NUBPL, NCKAP1, RPN1, SLC3A2, SLC7A11, ACTN4, ACTB, CD2AP, CAPZB, DSTN, FLNA, FLNB, INF2, IQGAP1, MYH10, MYL6, MYH9, PDLIM1, and TLN1.^12^-^14^

### Identification of lncRNAs associated with disulfidptosis:

We extracted the expression of disulfidptosis genes and performed co-expression analysis to predict the disulfidptosis-related lncRNAs, with the filtering conditions of r>0.4 and *P*<0.001. Then, we plotted a diagram using the “ggplot” package on the basis of the co-expression analyses. Lastly, we combined the expression data and survival data of the screened disulfidptosis-related lncRNAs.

### Construction of prognostic prediction model for disulfidptosis-related lncRNAs:

The LUAD clinical data were divided into training group and test group. Among the test set, univariate Cox regression analysis was utilized to evaluate the disulfidptosis-related lncRNAs. “glmnet” package was utilized to conduct LASSO Cox regression analysis and identify significant lncRNAs associated with disulfidptosis. Further screening of lncRNAs were performed through multivariate Cox regression. Then we built a prognostic prediction model and calculated the corresponding risk score.

### Efficacy analysis of prognostic prediction model:

According to the median risk value, we separated the samples into high and low risk groups. The “survival” package was utilized to conduct survival analyses on both groups of three data sets. We assessed the predictive performance of the model by utilizing receiver operating characteristic (ROC) curves and calculating the concordance index (C-index).

### Differential analysis of risk:

We read the samples from high and low risk groups and performed differential analysis, with screening criteria of logFC>1 and FDR<0.05. Results were utilized to perform gene ontology (GO) enrichment analysis and Kyoto Encyclopedia of Genes and Genomes (KEGG) pathway analysis. “GSEA” package was conducted to perform enrichment analysis on two groups and obtained the five most significantly enriched pathways.

### Analysis on tumor microenvironment:

We read the expression data and applied the “estimate” package to score the tumor microenvironment of each sample. The “ggpubr” package was utilized to draw differential analysis violin plot. CIBERSORT was utilized to calculate the infiltration abundance of twenty-two types of immune cells, containing T cells, naïve cells, memory B cells, plasma cells and NK cells, in the complex mixture of LUAD patient’s genes expression data. Wilcox test was employed to assess the microenvironment differences of disulfidptosis prognostic genes in the high- and low- risk groups.

## RESULTS

### Data retrieval and collation results:

Data of 600 tissue samples were obtained from TCGA, including 541 LUAD samples and 59 normal samples. Clinical data of 522 patients were acquired from TCGA, from which we extracted the patients’ data of age, gender, pathological stage, TNM stage, survival time and state of life. Filtration was employed if there were missing values, and finally we obtained 507 clinical data. We isolated the matrix containing RNA expression data by utilizing Perl software and obtained 19938 mRNAs and 16876 lncRNAs.

### Screening of disulfidptosis-related lncRNAs:

We retrieved 24 disulfidptosis-related genes from published literature and performed Pearson correlation analysis to investigate the association between lncRNAs and these genes in order to screen out lncRNAs.

### Establishment of prognostic prediction model:

Five hundred and seven patients were randomized into a training set of 254 patients and a test set of 253 patients. There was no significant variation between the data of the training set and the test set (P>0.05). We obtained from the literature that mRNAs of these 24 genes all expressed in LUAD tissues. Co-expression analysis between mRNAs and lncRNAs identified 127 disulfidptosis-related lncRNAs. The univariate Cox regression analysis was utilized to filter 11 lncRNAs which were related to LUAD prognosis. Further screening of 10 lncRNAs were performed through LASSO Cox regression. Finally, we utilized multivariate Cox regression analysis and screened out five valuable lncRNAs.

### Results of efficacy analysis of prognostic prediction models:

**Fig.1:**
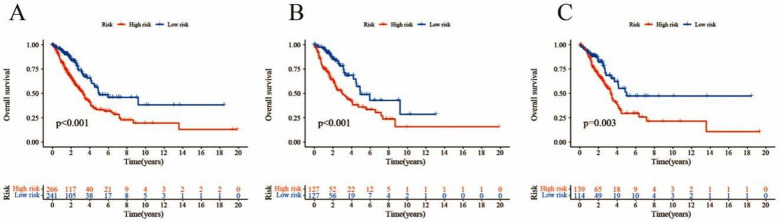
Survival analysis (A: all samples B: the training set C: the test set).

The survival analysis results indicated that the overall survival of the high-risk group in the three datasets was shorter than that of the low-risk group, and the progression-free survival (PFS) was also shorter, with statistical significance (P<0.05, Fig.1). In the training set, there was an increase in the number of patient deaths as the risk score increased, and this pattern was similarly observed and validated in the test set. Independent prognostic analysis indicated that the risk model and tumor stage may be prognostic. The area under the curve (AUC) for predicting the one years, three years, and five years survival rates using the risk model were 0.698, 0.658, and 0.595, respectively. Nomogram, combined with calibration curves, indicated that the predictive survival rate closely aligned with the announced survival rate.

### Differential analyses:

**Fig.2:**
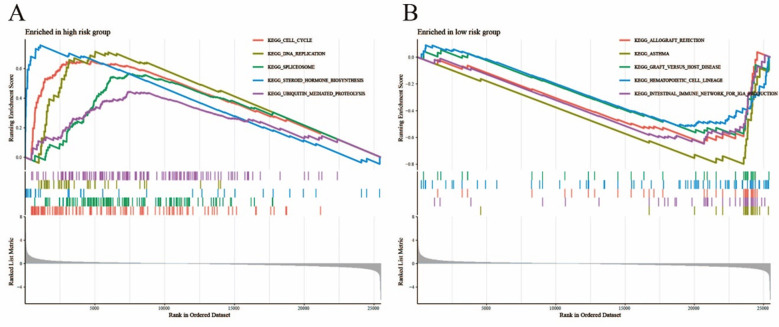
GSEA analysis (A: high-risk group B: low-risk group).

**Fig.3:**
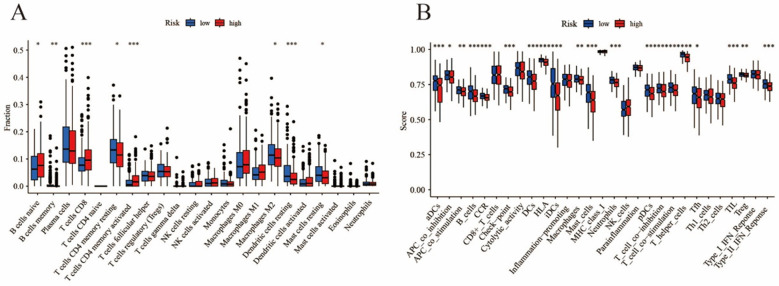
Analyses of immune cell differences (A: differential ana lyses of cell B: differential analyses of cell functions).

The GSEA analysis results were shown illustrated within Fig.2, among which the enrichment analysis of high-risk group contained cell cycle pathway and steroid hormone biosynthesis pathway, moreover, the enrichment analysis of low-risk group contained hematopoietic cell pathway and allograft rejection pathway. Analyses of cell differences and differential analyses of immune-related functions were shown in Fig.3.

## DISCUSSION

In this study we develop a LUAD prediction model of the disulfidptosis-related lncRNAs based on TCGA data and analyses utility of this model from a clinical perspective, which could help to provide new ideas and directions for the early clinical prevention of LUAD and theoretical foundation for the clinical management of LUAD. A recent study explored the important role of disulfidptosis-related lncRNAs in pan-cancer.^15^ Compared to this study, our study further explored the prognostic effect of disulfidptosis-related lncRNAs in LUAD.

Disulfidptosis is a recently discovered form of cell death. It is characterized as a rapid type of cell death triggered by the excessive buildup of cysteine, resulting in disulfide stress, which typically occurs under glucose starvation conditions. LncRNAs play crucial roles in regulating cell growth, metabolism, and can directly affect tumor cell proliferation, migration, invasion, apoptosis, and drug sensitivity.^16^ This study aimed to investigate the correlation between disulfidptosis-related LncRNAs and prognosis in LUAD based on the TCGA database. By analyzing data from the TCGA database, we utilized Lasso regression and Cox regression analysis to identify five LncRNAs and constructed a prognostic risk model.

Through survival analysis and ROC curve validation, showcased exceptional precision and dependability in forecasting the prognosis of LUAD patients. Furthermore, our prognostic model incorporates clinical staging and risk scoring and plays a good predictive role. The study results demonstrate that patients in the high-risk group have significantly lower overall survival, and the accuracy of the model has been validated through calibration curves and C-index. This indicates that our constructed model outperforms other clinical variables in terms of its predictive ability of survival time of LUAD patients and that the risk score is linked to the progression of LUAD.

In this study, the immune cell composition of two patient groups was evaluated using the CIBERSORT database. Research has shown that dendritic cells may potentially contributing to presenting tumor antigens to CD4 T cells, thereby promoting an anti-tumor immune response.^17^ Likewise, CD4+ memory T cells can recognize tumor cells and mount an immune response against them.^18^ Research has suggested that mast cells may secrete factors that promote tumor growth and metastasis, as well as factors that inhibit the anti-tumor immune response.^19^

On the other hand, high-risk group patients tended to be in an immune-activated state, including T cells CD4+ memory activated and T cells CD8+. Specific antigens expressed by cancer cells can be recognized by CD4+ memory activated T cells.^18^ CD8+ T cells can recognize and directly kill cancer cells by inducing apoptosis or cell death.^20^ The results indicate that patients categorized under the high-risk group are likely to exhibit a more favorable response to immunotherapy as compared to those in the low-risk group who may benefit from alternative therapeutic options. Consequently, the disulfidptosis-related LncRNA prognosis model has the potential to predict the effectiveness of immunotherapy for LUAD patients.

We have presented a prognostic model for LUAD using disulfidptosis-related lncRNAs, which could provide new directions and ideas for the diagnosis and treatment of LUAD. The theoretical significance of this study was undeniable, which had the potential to generate significant interest in the medical community and make more researchers pay attention to this direction. This innovative approach not only improved our understanding of the molecular mechanisms of LUAD, but also provided a new framework for strategies for cancer therapy and personalized treatment.

### Limitations

Further validation of the model is still required in larger clinical studies. These efforts are necessary to enhance the precision and dependability of the prognostic model. Experiments should be conducted to validate the results of our study. However, more studies are still needed to further analyze the prognostic effects of the LUAD lncRNAs risk model.

## CONCLUSION

The construction of a disulfidptosis-related LncRNAs prognosis model for LUAD shows potential for evaluating patient prognosis. This model can serve as a foundation for clinicians to evaluate prognosis and formulate personalized treatment plans based on individual risk levels.

## Data availability:

The data used and analyzed during the current research was available from the corresponding author at proper request.

### Author contributions:

**JL:** Contributed to the conception, design, manuscript writing, Data collection, conceptualization, and responsible for the integrity of the work. **WD:** Contributed to the conception, design, manuscript writing, data analysis, and interpretation of methodology. **HN:** Contributed to the design and manuscript writing. **WS:** Contributed to revised the manuscript and checked the accuracy or integrity of the work. All authors reviewed the manuscript.
